# Hybrid PET Track-Etched Membranes Grafted by Well-Defined Poly(2-(dimethylamino)ethyl methacrylate) Brushes and Loaded with Silver Nanoparticles for the Removal of As(III)

**DOI:** 10.3390/polym14194026

**Published:** 2022-09-26

**Authors:** Nursanat Parmanbek, Duygu S. Sütekin, Murat Barsbay, Anastassiya A. Mashentseva, Dmitriy A. Zheltov, Nurgulim A. Aimanova, Zhanar Ye. Jakupova, Maxim V. Zdorovets

**Affiliations:** 1 The Institute of Nuclear Physics of the Republic of Kazakhstan, Almaty 050032, Kazakhstan; 2 Department of Chemistry, L.N. Gumilyov Eurasian National University, Nur-Sultan 010008, Kazakhstan; 3 Department of Chemistry, Hacettepe University, Ankara 06800, Turkey; 4 Engineering Profile Laboratory, L.N. Gumilyov Eurasian National University, Nur-Sultan 010008, Kazakhstan

**Keywords:** composite track-etched membranes, reversible addition-fragmentation chain transfer polymerization, poly(2-(dimethyamino)ethyl methacrylate), silver nanoparticles, arsenic(III) removal, sorbent

## Abstract

Nanoporous track-etched membranes (TeM) are promising materials as adsorbents to remove toxic pollutants, but control over the pore diameter and density in addition to precise functionalization of nanochannels is crucial for controlling the surface area and efficiency of TeMs. This study reported the synthesis of functionalized PET TeMs as high-capacity sorbents for the removal of trivalent arsenic, As(III), which is more mobile and about 60 times more toxic than As(V). Nanochannels of PET-TeMs were functionalized by UV-initiated reversible addition fragmentation chain transfer (RAFT)-mediated grafting of 2-(dimethyamino)ethyl methacrylate (DMAEMA), allowing precise control of the degree of grafting and graft lengths within the nanochannels. Ag NPs were then loaded onto PDMAEMA-*g*-PET to provide a hybrid sorbent for As(III) removal. The As(III) removal efficiency of Ag@PDMAEMA-*g*-PET, PDMAEMA-*g*-PET, and pristine PET TeM was compared by adsorption kinetics studies at various pH and sorption times. The adsorption of As(III) by Ag@DMAEMA-*g*-PET and DMAEMA-*g*-PET TeMs was found to follow the Freundlich mechanism and a pseudo-second-order kinetic model. After 10 h, As(III) removal efficiencies were 85.6% and 56% for Ag@PDMAEMA-*g*-PET and PDMAEMA-*g*-PET, respectively, while PET template had a very low arsenic sorption capacity of 17.5% at optimal pH of 4.0, indicating that both PDMAEMA grafting and Ag-NPs loading significantly increased the As(III) removal capacity of PET-TeMs.

## 1. Introduction

As an extremely toxic element to human health and the environment, arsenic is quite ubiquitous based on both natural and anthropogenic sources. It is naturally present at high levels in the groundwater of a number of countries and estimates put the number of people poisoned by arsenic in the hundreds of millions mainly due to increased groundwater uptake, perhaps the largest poison in world history [1,2]. The application of functional materials with high surface area and multiple uses is rapidly growing in addressing this global health and environmental concern.

The development of tailor-made membranes in which mass transfer and phase boundary properties can be regulated can create practical and effective alternatives for removing water contaminants, including arsenic [3,4]. Membranes with controllable high roughness, such as nanoporous track etched membranes (TeMs), are of particular interest, since unclogged and effectively functioning nanochannels can work locally at high rates and with high selectivity. TeMs have a unique pore structure that offers the smallest tolerances regarding pore diameter and density compared with alternative porous membranes in the literature. The two-step process applied in their preparation (bombardment with accelerated heavy ions and subsequent chemical etching) allows them to precisely target specific requirements regarding the size and density of the nanochannels, which affect surface area, a key focus of absorption studies [5,6,7].

Grafting of polymers on TeMs has become a common practice for obtaining specific chemical functionalities inside the nanochannels [8]. Most of the surface properties of materials are determined by the phase at the boundary between the substrate and the environment. Surface modification controls the boundary properties such as adhesion, wettability, protection against corrosion, ion-exchange, and biocompatibility to withdraw the attention of many application areas such as biomedical, automobile, lubrication, and depollution. One of the most effective methods used by researchers to obtain materials with new surface properties is graft copolymerization [9,10,11]. This technique is commonly used because it builds a robust chemical bonding between the substrate and the new functional layer. Although conventional graft copolymerization is very effective in changing the surface properties, it is quite insufficient in making this change in a controlled way. It is not possible to adjust a number of properties, particularly the molecular weights and architectures of polymer chains grafted to substrate via the conventional free radical graft copolymerization techniques [9,12,13]. Especially in the case of confined spaces such as nano-sized channels, as with TeM’s, using traditional grafting methods can lead to their plugging, which means losing the most basic advantage, i.e., high surface area, from the beginning [12]. Incorporation of reversible-deactivation radical polymerization (RDRP) techniques to nanochannel functionalization by grafting is promising as these techniques allow precise control of the degree of grafting and graft lengths within the nanochannels so that they do not block the pores for subsequent applications. RDRP methods such as atom transfer radical polymerization [13,14] and reversible addition fragmentation chain transfer polymerization (RAFT) [15,16] have been successfully applied in nanochannel grafting in a controlled manner.

In light of the foregoing, in this study we combined different strategies to develop a high-capacity sorbent membrane for the removal of trivalent arsenic, As(III), which is a major drinking-water contaminant. The toxicity and health hazards of arsenic are well-known [17,18]. Due to the high affinity of arsenic for protein, both trivalent and pentavalent forms easily accumulate in living tissues [19]. The lower-oxidation-state form, i.e., As(III), is more mobile and about 60 times more toxic than As(V) [20]. In groundwater, As(III) is present as H_3_AsO_3_, H_2_ASO_3_^−^, and HAsO_3_^2−^, which are not efficiently absorbed by minerals, while As(V) is readily absorbed onto solid mineral surfaces [21]. Due to its higher toxicity and mobility, further research is required to remove As(III). It is a well-known method to use polymers, such as poly(2-(dimethyamino)ethyl methacrylate) (PDMAEMA), to remove arsenic from aqueous media due to their high sorption capacity [22,23]. PDMAEMA is a water-soluble cationic polymer with tertiary amine functional groups prone to complexation with arsenic. Another approach to remove fairly high amounts of As(III) is to use nanoparticles (NPs) of metal oxides [24], such as TiO_2_ [25,26,27], Fe_2_O_3_ [28], Fe_3_O_4_ [29,30], CeO_2_ [31], CeO_2_-ZrO_2_ [32], CuO [33], Cu/CuO [34,35], Al_2_O_3_ [36], ZrO_2_ [37], and CaO_2_ [38]. Zerovalent metal NPs such iron NPs [39], palladium NPs [40], and silver (Ag) NPs [41,42] have also been shown to be effective in As(III) removal. Besides being effective in the removal of other contaminants such as Co(II) and Pb(II) [43], Ag NPs are also notable for being widely used in drinking water disinfection due to their excellent bactericidal performance [44]. A recent study by Mukherjee et al. showed, not only through the results of atomic absorption spectroscopy but also by several biophysical techniques as well as interaction studies, that monolayer protected Ag NPs efficiently remove As(III). The adsorption capacity reported (4.69 mg/g) was superior to some well-known nanoadsorbents such as maghemite (γ-Fe_2_O_3_) and zero-valent iron [41].

**Figure 1:**
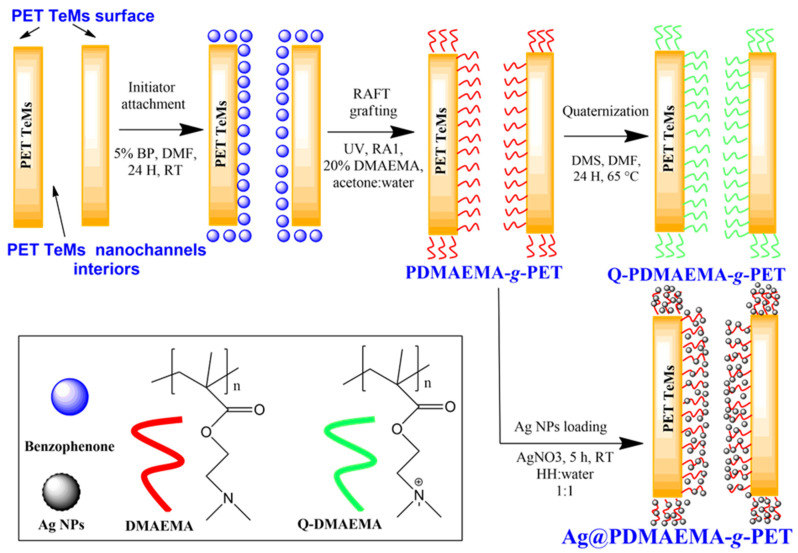
Simplified scheme for the preparation of hybrid track-etched membranes grafted by well-defined poly(2-(dimethylamino)ethyl methacrylate) brushes and loaded with silver nanoparticles.

From the above discussion, an urge motivates us to use Ag NPs simultaneously with a well-performing polymer adsorbent, i.e., PDMAEMA to remove arsenic from aqueous media. The present paper firstly reported on the combination of RAFT polymerization and graft copolymerization to functionalize the entire surface and interior of the nanochannels of track-etched PET membrane with PDMAEMA. The well-defined PDMAEMA chains grown by RAFT mediated grafting from the etched nanochannel walls and surface of the PET membrane via the UV-activation of a photoactive tethering reagent, i.e., benzophenone moiety, immobilized to the whole surface provided a functional absorbent surface towards As(III) ions and also stabilized the Ag NPs to be loaded in the next step, as schematically illustrated in Figure 1. Entire PET TeM surfaces functionalized both by PDMAEMA and Ag NPs served as a hybrid sorbent for the removal of As(III).

## 2. Materials and Methods

### 2.1. Materials

Benzophenone (BP), ethanol, methanol, sodium hydroxide (NaOH), hydrogen peroxide (H_2_O_2_), 2-(dimethyamino) ethyl methacrylate (DMAEMA), hydrochloric acid (HCl), hydrazine hydrate, potassium ethyl xanthogenate (PEX, 96%), and methyl-2-bromopropionate (MB, 98%) were purchased from Sigma Aldrich (Schnelldorf, Germany). DMAEMA was passed through an alumina column to remove the polymerization inhibitor. O-ethyl-S-(1-methoxycarbonyl) ethyl dithiocarbonate (RA1) was applied as the RAFT agent and synthesized according to the literature using PEX and MB (yield: 95.1%) [45].

The certified reference solution at a concentration of 0.1 g/L As(III) was purchased from Ecroskhim (Sankt-Petersburg, Russia). The water used in all the experiments was purified using a “Aquilon—D301” water purification system with a resistivity of 18.2 MΩ/cm (Aquilon, Podolsk, Russia).

### 2.2. Irradiation and Track-Etching of PET Films

To obtain polymer templates, a PET Hostaphan^®^ RNK film (film thickness is 12.0 microns) was irradiated by ^84^Kr^15+^ ions with 1.75 MeV/nucleon energy and 4.2 × 10^7^ ion/cm^2^ fluency (Cyclotron DC-60, Institute of Nuclear Physics of Kazakhstan) and then etched in 2.2 M NaOH. At the end, PET TeMs with an average pore diameter of 385 ± 9 nm were attained. Samples were kept in air at room temperature.

### 2.3. Grafting of PDMAEMA from the Nanochannels of PET TeMs (PDMAEMA-g-TeMs)

Benzophenone (BP) is frequently used as a photoactive tethering reagent to functionalize the surface of various materials, including commercial plastics and fabrics [46,47,48]. There is a straightforward relationship between the immobilized BP concentration on a surface and grafting yield. In order to increase the amount of BP immobilized on the PET TeMs, oxidation of the substrate, thus increasing the carboxyl group concentration on the surface, has been reported as an effective strategy [49,50,51]. Therefore, PET TeM films were first oxidized prior to UV-induced grafting experiments. In the oxidation step, pristine PET TeMs were treated with a 500 mM H_2_O_2_ solution at pH 3 for 180 min under UV irradiation (190 W at 254 nm) [52]. The samples were then washed twice with deionized water and air-dried at room temperature for 5 h. The amount of -COOH groups was determined via a titration method and was determined as 18.17 ± 3.2 nmol/cm^2^ (average of five measurements). For the immobilization of BP, the oxidized PET TeMs were soaked in 5% BP in DMF (*w*/*v*) and kept in a shaking water bath at 150 rpm for 24 h at room temperature. The membranes on which BP was physically attached were then washed with water and ethanol, dried, and rapidly used for the grafting experiments.

Grafting of DMAEMA from the BP-immobilized PET TeMs was carried out in a controlled manner by RAFT polymerization using two molar ratios of DMAEMA and the xanthate-based RA1 RAFT agent ([DMAEMA]/[RA1] = 500, 1000). The concentration of DMAEMA was varied as 5%, 10%, 20%, 30%, and 40% (*w*/*v*) in a total solution volume of 10.0 mL. The solutions were prepared using four different solvents, i.e., water, acetone:water (1:1, *v*/*v*), ethanol:water (1:1, *v*/*v*), and ethanol. Reaction solutions were put into sealed flasks containing BP-immobilized TeMs and degassed with argon for 10 min prior to polymerization. Two different polymerizations, thermal and UV-initiated, were examined. For thermal polymerizations, a water-soluble thermal initiator, namely 4,4′-Azobis-4-cyanopentanoic acid (ACPA), was also added to reaction medium and the solutions were placed in a shaking water bath (150 rpm, IKA KS 3000 IS control, (IKA, Konigswinter, Germany)) at 70 °C for 60 to 1440 min. For UV-assisted graft copolymerization, samples were placed under a UV-lamp (15 W at 295 nm, Ultra-Vitalux 300 W, Osram, Augsburg, Germany) at a distance of 7 cm where the reaction time was varied from 30 to 1440 min. After each predetermined grafting period, the grafted films (PDMAEMA-*g*-TeMs) were taken from the reaction medium and washed with deionized water by periodically changing the solvent. Degree of grafting (DG) was determined from initial and final weights obtained using an analytical balance (Mettler Toledo, Columbus, OH, USA) and calculated with an accuracy of ±0.05 mg using the Equation (1):

(1) $$DG=\frac{\left(w_{f}-w_{i}\right)}{w_{i}}\times 100\%$$

where, *w_f_* is the weight of the PDMAEMA-*g*-TeMs films and *w_i_* is the weight of the BP-immobilized PET TeMs.

### 2.4. Quaternization of PDMAEMA-g-TeMs (Q-PDMAEMA-g-TeMs)

According to the procedure described elsewhere [53], samples of PDMAEMA-*g*-TeMs were immersed in dimethyl sulfate (DMS) solution in DMF and heated in water bath at 65 °C for 24 h with stirring. After that, the samples were removed from reaction medium and washed with DMF, water, and methanol several times and dried at room temperature. The degree of quaternization for Q-PDMAEMA-*g*-TeMs samples, which describes the amount of nitrogen atoms quaternized, was calculated from the mass increment after the quaternization process. Approximately 90% quaternization was achieved in 24 h.

### 2.5. Loading of Ag NPs onto PDMAEMA-g-TeMs (Ag@PDMAEMA-g-TeMs)

Prior to electroless plating of silver, PDMAEMA-*g*-TeMs were immersed in a saturated solution containing AgNO_3_ in order to adsorb silver ions on the surface of the sample. This procedure was carried out at the temperature of 25 °C for 5 h in a shaker (130 rpm, IKA KS 3000 IS control, (IKA, Konigswinter, Germany). Afterward, samples were removed from saturated solution of silver nitrate and reduced by hydrazine hydrate (1:1 with deionized water). The amount of silver deposited was determined gravimetrically based on the difference in the weights of the composite before and after plating with an accuracy of 0.1 mg (AS 220.R2, Radwag, Radom, Poland) and expressed in units of mg/cm^2^.

### 2.6. Batch Absorption Experiments

All experiments conducted to determine the As(III) adsorption performance of TeMs were carried out using batch equilibrium techniques. Feed As(III) solution (100 ppm, pH 4.0) was prepared by diluting the certified As(III) reference solution (0.1 g/L, Ecroskhim, Russia). Adsorption kinetics were studied at an As(III) concentration of 50 µg/L (pH 4.0). Disposable plastic vials (Isolab, Eschau, Germany) containing 15.0 mL of solution and 2 × 2 cm of composite adsorbate were shaken (100 rpm, IKA KS 3000 IS control, (IKA, Konigswinter, Germany) at room temperature for different times between 15 min and 10 h. Each experiment was repeated in triplicate. The concentration of As(III) in aliquots was determined by ICP–MS (Thermo Fisher Scientific, XSeries 2, Bremen, Germany). The adsorbed amount of As(III) was calculated using Equation (2) [34]:

(2) $$Q_{e}=\frac{\left(C_{0}-C_{e}\right)\times V}{m}$$

where *Q_e_* is the amount of As(III) adsorbed by the unit mass of TeMs (mg/g), *C*_0_ is the feed concentration (mg/L), *C_e_* is the concentration of As(III) in aliquots (mg/L), *V* is the volume of the solution (L), and *m* is the amount of silver loaded on the membrane used (g). In the case where the pristine template was tested, the weight of PDMAEMA-*g*-TeMs and Q-PDMAEMA-*g*-TeMs were used in *m* (g).

The effect of pH on As(III) adsorption was studied in the pH range of 3 to 9. Other parameters were kept constant (initial As(III) concentration: 50 ppm; adsorbent dose: 2 × 2 cm^2^; contact time: 300 min). The pH of the solution was adjusted dropwise with 1.0 N HCl_(aq)_ and 1.0 N NaOH_(aq)_. The pH was measured using a digital pH meter, HANNA HI2020-02 (HANNA Instruments, Smithfield, UT, USA). All experiments were performed in triplicate.

The charge on the adsorbent surface depending on the pH value was studied by determining the pH_zpc_ value in the pH range from 3.0 to 9.0 according to the method described in ref. [54]: 10 mL of NaCl solution (0.01 M.) was brought to the desired pH value (pHi) by adding 0.1 M. of HCl or NaOH. After that, sample with the size of 2 cm × 2 cm was added to each flask and shaken on a shaker IKA KS 3000i (IKA, Konigswinter, Germany) for 12 h at room temperature and the final pH (pH_f_) of the filtrate was measured using HANNA HI2020-02 pH-meter (HANNA Instruments, Smithfield, Smithfield, UT, United States of America).

### 2.7. Characterizations

PET TeM films were visualized by environmental scanning electron microscopy (SEM, FEI Quanta 200F ESEM, ThermoFisher Scientific, Hillsboro, OR, USA) operating at 10 kV. The elemental composition of the composites was studied by a Hitachi TM3030 SEM (Hitachi Ltd., Chiyoda, Tokyo, Japan) equipped with a Bruker XFlash MIN SVE (Bruker, Karlsruhe, Germany) microanalysis system at an accelerating voltage of 15 kV.

Attenuated Total Reflectance Fourier Transform Infra-Red (ATR-FTIR) spectroscopy measurements were carried out in ATR mode using a Spectrum One FTIR spectrometer (Perkin Elmer, Waltham, MA, USA). Each spectrum was obtained in the wave number range of 4000–450 cm^−1^, with a discrimination of 4.00 cm^−1^, from 32 scans.

The crystal structure of the nanoparticles was examined on a D8 Advance diffractometer (Bruker, Karlsruhe, Germany) in the angular range of 2θ 30–80° with a step of 2θ = 0.02° (measuring time: 1 s, tube mode: 40 kV, 40 mA). The mean size of crystallites was determined via the broadening of X-ray diffraction reflections using the Scherer equation [55]. The phase composition was determined using the Rietveld method, which is based on approximating the areas of the diffraction peaks and determining the convergence with reference values for each phase [34]. The volume fraction of the composite phase was determined using Equation (3):

(3) $$V_{admixture}=\frac{RI_{phase}}{I_{admixture}+RI_{phase}},$$

where *I_phase_* is the average integral intensity of the main phase of the diffraction line, *I_admixture_* is the average integral intensity of the additional phase, and *R* is the structural coefficient equal to 1.

XPS measurements were carried out using a Thermo Scientific K-Alpha spectrometer (Waltham, MA, USA) with a monochromatized Al Kα X-ray source (1486.6 eV photons) at a constant dwell time of 100 ms, pass energy of 30 eV with a step of 0.1 eV for core-level spectra and 200 eV with a step of 1.0 eV for survey spectra. The pressure in the analysis chamber was maintained at 2·10^−9^ Torr or lower. All samples were analyzed at a take-off angle of 90°. Surface elemental composition was determined using an X-ray spot size of 400 μm by varying the energy between 0 and 1000 eV. Binding energies (BEs) were referenced to the C1s hydrocarbon peak at 285 eV. Processing of the data was carried out using Avantage software (version 5.41, 2019, Waltham, MA, USA).

The pore size of the pristine and grafted TeMs and the structural parameters of the composites obtained were determined by porometry using the Hagen–Poiseuille equation [7]. X-ray diffraction (XRD) patterns were obtained on a D8 Advance diffractometer (Bruker, Karlsruhe, Germany) to study the crystalline structure of the samples. X-ray was generated at 25 mA and 40 kV, and the scanning position ranged from 30° to 90° 2(θ). The average crystallite size was determined using the Scherrer equation [56].

The surface morphology of the pristine, grafted, and composite membranes was studied by a scanning probe microscope (SmartSPM-1000, NT-MDT, Novato, CA, USA) in semicontact mode using an NSG10 (TipsNano, Tallinn, Estonia) rectangular-shaped silicon cantilever (length, 95 ± 5 μm; width, 30 ± 5 μm; thickness, 1.5–2.5 μm; probe tip radius, 10 nm; resonance frequency, 200 kHz). Initial scanning of a 10 × 10 μm^2^ sample was performed at a speed of 5.0 μm/s. The average roughness was calculated from a 3 × 3 μm^2^ scanning area. The data obtained were processed and analyzed by using the IAPRO-3.2.2 software.

The water contact angle (CA) values of oxidized and PDEMAEMA grafted PET TeM surfaces were measured at ambient temperature using a DSA-100 goniometer system (Krüss Company, Hamburg, Germany). The average CA was obtained by at least three repetitive measurements by 10 μL drop volume of deionized water using the Young–Laplace method of the Drop Shape Analysis program (Krüss Company, Hamburg, Germany).

Thermal properties of polymers were recorded using a Perkin–Elmer thermogravimetric analyzer Pyris 1 TGA (Perkin Elmer, Waltham, MA, USA). Analyses were conducted over the temperature range from 30 to 600 °C with a programmed temperature increment of 25 °C min^−1^ under N_2_ atmosphere.

## 3. Results

### 3.1. Characterization of the Composite Membranes

Polymers synthesized by RDRP techniques possess well-defined molecular architectures and are used in many applications such as drug-delivery [57], sensors [58,59], molecular imprinting [60,61], polymer–protein conjugates, development of cylindrical, spherical, hyper-branched polymers, pH or temperature responding smart polymers, etc. [62]. These studies prove that the versatile RDRP techniques can meet the requirements of highly functional complex polymeric architectures that have the advantage of well-defined and controllable properties. Among the parameters that determine the success of the RAFT mechanism in controlling the polymer architecture, the selection of the RAFT agent is particularly important. Xanthate-type RAFT agents (dithiocarbonates) have emerged as functional chain transfer agents (CTAs) mostly used for the controlled synthesis of less-activated monomers containing a double bond adjacent to an electron-withdrawing group such as nitrogen, oxygen, or halogen atom. The RAFT polymerization of DMAEMA can be successfully carried out using a xanthate such as RA1 [63]. Grafting of monomers on BP-immobilized PET substrate can be carried out thermally [64] or by UV-initiation [50]. Activating BP thermally, instead of photochemically, is promising because most of the common RAFT agents have low resistance against UV-radiation. Therefore, we first carried out the grafting of DMAEMA by thermally initiated protocol. Unfortunately, the highest DG attained at almost 24 h was only 2.4% at a DMAEMA feed concentration of 10% (*v*/*v*). This low grafting rate is not satisfying for many applications. Therefore, confirming the UV-stability of the RAFT agent used (Supplementary Information, Figure S1a), we decided to carry out the grafting under UV irradiation. It is well documented that the photochemically generated triplet state of BP is able to abstract a labile hydrogen atom from almost all polymers, which causes the formation of high concentrations of radicals on the substrate surface [65]. The resulting radicals on the surface combine with monomers in the polymerization medium and graft copolymerization takes place over the entire surface area where BP generates radicals, including the nanochannel interiors, as schematically illustrated in Figure 1 [65,66]. This photoinduced grafting method has been used to covalently attach various polymers to a wide range of substrates for many different applications such as sensors [64], lithography [67], biosensors [68], organic semiconductors [69], etc.

**Figure 2:**
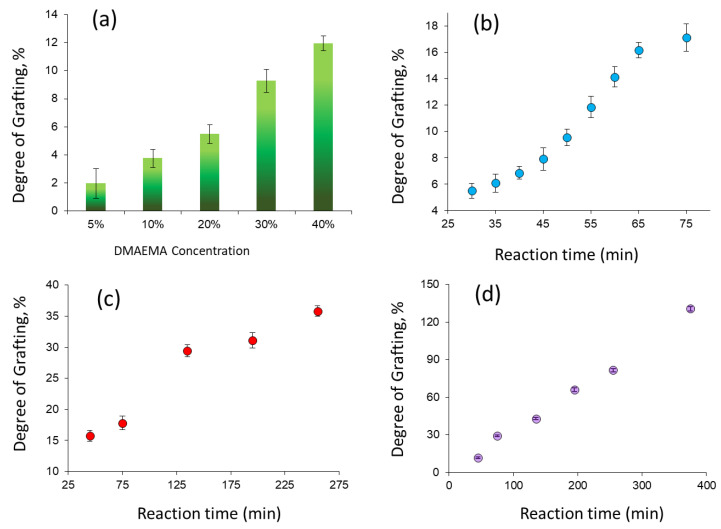
Effect of monomer concentration (t = 50 min) (**a**) and reaction time on the UV-initiated, RAFT-mediated grafting of DMAEMA (20%, *v*/*v*) from PET-TeMs using [DMAEMA]/[RA1] molar ration of 500/1 in water (**b**). Effect of reaction time on the UV-initiated, RAFT-mediated grafting of DMAEMA (20%, *v*/*v*) from PET-TeMs using [DMAEMA]/[RA1] molar ratio of 1000/1 in water (**c**) and in acetone:water (1:1) (**d**).

UV-initiated grafting of DMAEMA was carried out by varying monomer concentration, reaction time, and solvent choice as summarized in Table S1, Supplementary Information. As a result of the polymerizations carried out in water, the desired amount of grafting was obtained by changing the reaction time or monomer concentration, as seen in Figure 2a,b and Table S1. However, a rapid gel formation and precipitation was observed during polymerizations in water. Even after 30 min of UV irradiation, the solution became cloudy (see Figure S1b, Supplementary Information), which is an obstacle to achieving the structural control expected from RAFT polymerization mechanism. In addition, the gelling observed in the aqueous medium can lead to the closure of the nanochannel inlets and the inability to graft their interiors, resulting in a heterogeneous grafting profile and possible performance losses in subsequent applications. It was observed that gelation did not occur in other solvents studied. Among them, the highest degree of grafting (DG) was obtained in the acetone-water (1:1, *v*/*v*) mixture as seen in Table S1, Supplementary Information (compare entries 5, 20–22).

**Figure 3:**
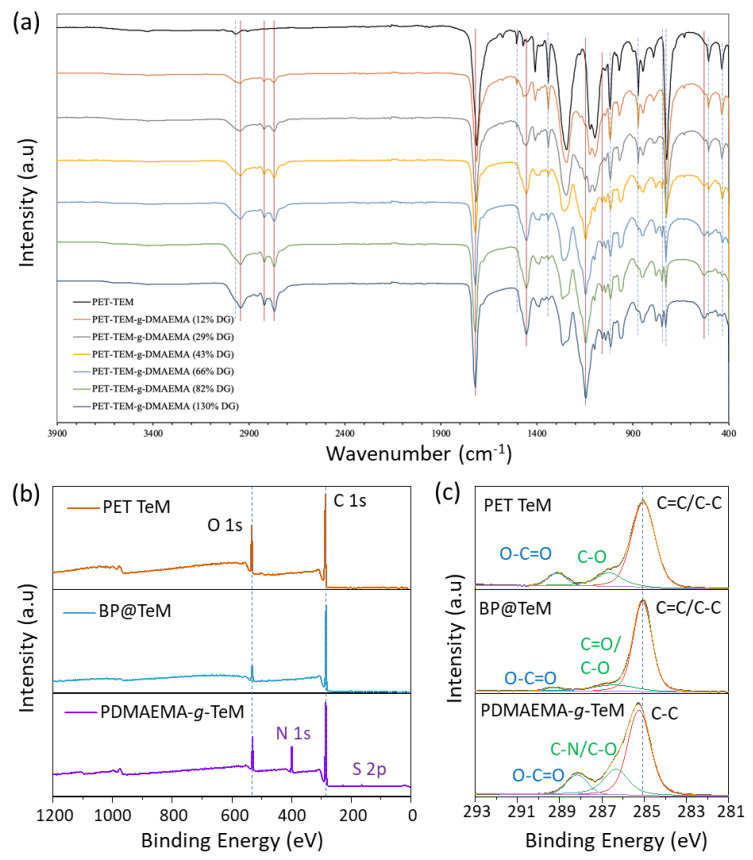
FTIR spectra of pristine PET TeM and PDMAEMA grafted membranes with different degrees of grafting (**a**). XPS survey wide scan spectra (**b**) and core-level C 1s spectra of PET TeM, BP immobilized PET TeM (BP@TeM), and PDMAEMA-*g*-TeM (DG: 12%) (**c**).

In the light of these results, acetone-water mixture and 20% (*v*/*v*) monomer concentration, where the desired level of grafting can be achieved without causing overheating of the polymerization solution under UV-irradiation, were chosen as the optimal conditions for further studies. Increasing the [DMAEMA]/[RA1] molar ratio leads to an increase in the targeted theoretical molecular mass of polymer chains and an acceleration of polymer kinetics, and thus an increase in grafting degree [45,70]. To further increase the DG, we reduced the amount of RAFT agent used by changing the [DMAEMA]/[RA1] molar ratio from 500/1 to 1000/1. As can be seen from entries 23 and beyond in Table S1, reducing the amount of RAFT agent resulted in an increase in DG, although other parameters were similar. Grafting of DMAEMA from PET-TeMs in water yielded DGs less than 35% for up to 255 min (Figure 2c), whereas higher DG values and moreover a linear trend were observed in the acetone-water mixture (Figure 2d), indicating the suitability of this solvent mixture for achieving high values and linearity in DG by reaction time and the presence of a controlled grafting fashion. Conventional grafting carried out in the absence of RAFT agent (entries 35–38, Table S1) resulted in much higher DG values compared with those mediated by the RAFT mechanism under the same experimental conditions. This is a clear indication that grafting of DMAEMA from PET TeMs proceeds in a controlled manner by the xanthate-based RAFT agent used, namely RA1. The chemical structures of TeM prior to the grafting and PDMAEMA-*g*-PeM samples with different DGs were evaluated using FTIR spectroscopy as shown in Figure 3a. Besides the characteristic peaks of PET structure (denoted as blue dashed lines), new characteristic absorption peaks of PDMAEMA (red lines) can be identified in the grafted samples. Grafting of PDMAEMA from TeMs significantly led to the appearance of the stretching vibrations of C-H in -N(CH_3_)_2_ moieties at around 2940, 2820, and 2760 cm^−1^. Carbonyl stretching vibration of the TeM was observed at 1712 cm^−1^ prior to grafting, while a slightly higher wavenumber (1720 cm^−1^) was observed after the grafting of PDMAEMA. In addition, the stretching vibration at 1145 cm^−1^ corresponding to C-N vibrations and -CH_2_ deformation peak of the PDMAEMA backbone at 1454 cm^−1^ were identified in the spectra of the grafted samples [71,72]. All the characteristic peaks of PDMAEMA became more distinct with increasing DG.

The chemical structures of TeM prior the grafting and PDMAEMA-*g*-PeM samples with different DGs were evaluated using FTIR spectroscopy as shown in Figure 3a. Besides the characteristic peaks of PET structure (denoted as black dashed lines), new characteristic absorption peaks of PDMAEMA (red lines) can be identified in grafted samples. Grafting of PDMAEMA from TeMs significantly led to the appearance of the stretching vibrations of C-H in -N(CH_3_)_2_ moieties at around 2940, 2820, and 2760 cm^−1^. Carbonyl stretching vibration of the TeM was observed at 1712 cm^−1^ prior to grafting, while a slightly higher wavenumber (1720 cm^−1^) was observed after the grafting of PDMAEMA. In addition, the stretching vibration at 1145 cm^−1^ corresponding to C-N vibrations and -CH_2_ deformation peak of the PDMAEMA backbone at 1454 cm^−1^ were identified in the spectra of the grafted samples [71,72]. All the characteristic peaks of PDMAEMA became more distinct with increasing DG.

In order to elucidate the composition and chemical environment of the samples along the surface extending from the top monolayer to a depth of about 10 nm, we carried out XPS analysis. Figure 3b shows the survey wide scans for photoelectrons emitted from oxidated, BP-immobilized, and PDMAEMA-grafted (DG: 12%) PET TeMs. The O/C molar ratio, calculated as 2.7/5 from XPS data for oxidized TeM, was higher than the theoretical value of 2.5 for PET, which is expected due to the oxidation on the surface. As a result of the immobilization of BP to the surface, this molar ratio decreased to approximately 1/9, confirming that the surface was enriched in carbon due to the immobilization of BP molecules on the topmost surface of PET. Following grafting of PDMAEMA, which is richer in oxygen compared with BP, this ratio increased in favor of O, as can be seen from the bottom spectrum in Figure 3b.

It is worth noting that the presence of N atoms in the structure of PDMAEMA causes a strong N 1s peak to appear around 400 eV. The C/N molar ratio of the grafted sample was 7.6/1, very close to the theoretical value of 8/1 for DMAEMA. This molar ratio and the overall appearance of the spectrum for the sample with a higher grafting degree (DG: 29%, data not presented here) were almost identical, indicating that a PDMAEMA layer of at least 10 nm thick had already covered the surface, in agreement with the SEM findings to be discussed below. In addition, a small contribution of S atoms in the spectrum of the grafted sample is remarkable because it corresponds to the RAFT chain-end moieties and indicates that the grafted PDMAEMA layer is grown through the RAFT mechanism. High-resolution C 1s core level photoelectron spectra of these the same samples, which clearly show the C atoms bound to another C or H, as well as those bound to hetero atoms (O or N), are given in Figure 3c. The previously reported binding energies of PET are in excellent agreement with our data [64]. The shape or position of the C peak components changes depending on the modification carried out. Due to the immobilization of BP, there has been a significant decrease in the amount of C species bound to O. The presence of O-C=O species emerging at about 289.5 eV is attributed to photoelectrons emitted from the PET substrate. After grafting, however, the surface was enriched in hetero atom-bounded species. In addition, a slight shift in the binding energy of C-C species is attributed to a completely different chemical environment, indicating the coverage of the surface by PDMAEMA layer.

**Figure 4:**
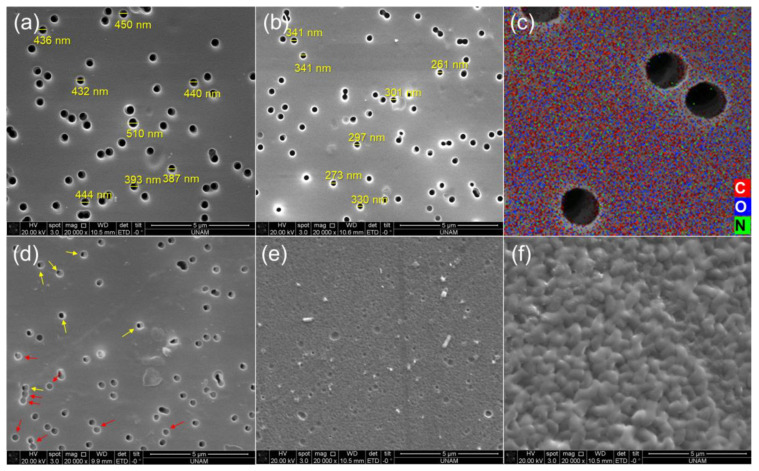
SEM image of pristine PET TeM (**a**), PDMAEMA-*g*-TeM (DG: 12%) (**b**), SEM-EDX elemental mapping for C (red), O (blue), and N (green) for PDMAEMA-*g*-TeM (DG: 12%) (**c**). SEM image of PDMAEMA-*g*-TeM (DG: 29%) (**d**), SEM image of PDMAEMA-*g*-TeM (DG: 35%) (**e**), and SEM image of PDMAEMA-*g*-TeM (DG: 22%) synthesized by a conventional method instead of RAFT-mediated grafting (**f**).

SEM was used to investigate the changes in the morphology and pore diameter of grafted membranes as DGs increase. Figure 4 shows both the evolution of the surfaces of pristine and PDMAEMA grafted nanoporous PET TeMs, along with their corresponding measured pore diameters.

For the ungrafted TeM, the pore diameter was about 437 ± 38 nm (Figure 4a) while the diameter of the PDMAEMA grafted membrane with 12% DG was about 306 ± 32 nm (Figure 4b) showing a decrease of about 130 nm in the diameter of the pores. The SEM-EDX atomic mapping image presented in Figure 4c for C, O, and N shows that these elements were uniformly distributed over the entire surface and that the surface was homogeneously covered with PDMAEMA, especially due to the presence and distribution of N atoms. As the immobilized BP is able to cleave the labile hydrogen atoms only on the topmost surface level, the created radials are on the surface and hence the growing PDMAEMA chains occurs only from the surface, and not through the inside the PET bulk. The increase in DG therefore leads to an increase in the thickness of the grafted PDMAEMA layer on the nanochannel walls. As can be seen by comparing Figure 4a,b,d,e, the pore diameter decreased as a function of DG, appraising gradual saturation of the nanochannels and reflecting the living/well-controlled character of the RAFT-mediated radical copolymerization between PDMAEMA grafts and the PET TeMS. In Figure 4e, no open pores were observed for the sample with DG of 35%, but the entries of some fully loaded pores were evident. This SEM image shows that the nanochannels were completely filled by the grafted PDMAEMA and the excess overflows from the pores giving crater-like formations. Similar formations and closure of the pores (red arrows) began to appear in the SEM image of the 29% PDMAEMA grafted membrane (Figure 4d), along with some still-open pores around a few tens of nm in diameter (yellow arrows). In the light of these observations, the optimal grafting degree was determined to be around 20%. From the SEM images, morphological changes are evident not only inside the nanochannels but across the entire surface of the TeMs. This SEM study allows visualizing at the nanoscale that the initiation of the polymerization takes place from the pore walls and the entire surface of the PET TeMs, as supported by the XPS analysis as well. Another striking result obtained from the SEM analysis is that, in the SEM image of the conventionally grafted sample (Figure 4f), unlike the RAFT-initiated grafting, it is seen that the surface completely covered by a PDMAEMA layer with irregular morphology and all the nanochannels were clogged at a grafting degree of 22%, indicating a non-gradual and uncontrolled grafting fashion.

**Figure 5:**
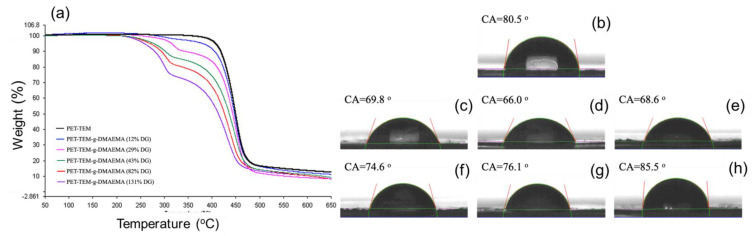
Thermogravimetric curves for pristine and PDMAEMA grafted PET TeMs heated in a 50–650 °C range (**a**)**.** Contact angle (CA) measurements of oxidized PET TeM (**b**) and PDMAEMA grafted TeMs with different DGs; 12% DG (**c**), 22% DG (**d**), 29% DG (**e**), 35% DG (**f**), and 43% DG (**g**), and 66% DG (**h**).

In addition to the structural characterizations, the study of the thermal behavior of the grafted samples indicated that the aforementioned modifications were carried out successfully. The TGA thermograms presented in Figure S1 and their comparison in Figure 5a show that in addition to the characteristic one-step thermal degradation profile of intact PET TeM observed between 327 and 534 °C with a mass loss of 84.9% [73], an additional degradation step appeared at approximately 200–350 °C range upon PDMAEMA grafting. This new degradation peak belongs to PDMAEMA [74], and its intensity increased with an increase in DG. The contact angle (CA) measurements provide important information about the changes occurring on the surface. The oxidized PET-TeM (Figure 5b) gave a CA of 80.5°, in accordance with the previous data [75]. It was observed that the CA decreased significantly from 80.5° to 76.1°, 69.8°, and 66.0° for PDMAEMA grafted membranes with 12%, 22%, and 29% DG, respectively, depending on the hydrophilic character of PDMAEMA grafts compared to PET substrate. However, the water droplet spread less on the surface beyond 29% grafting, yielding an increase in CA values. As can be seen from the digital images of PDMAEMA-grafted PET TEMs (Figure S2 of Supplementary Information), the surface character of the grafted membranes did not change significantly by DGs up to 29%, while it differed markedly for samples with higher grafting degrees. The observed increase in CA values of membranes with high DGs may be due to the increase in roughness [76]. Quaternization and Ag NPs loading processes also cause serious changes in CA. Due to the increased hydrophilicity after quaternization [77], the CA value of the membrane with 22% DG decreased from the initial value of 69.8° to 45.2° (Supplementary Information, Figure S3a,b). On the other hand, following the deposition of Ag NPs to the membranes, there was a very significant increase in CA, mainly due to increased porosity, in agreement with previous reports [78,79]. As can be seen from Figure S3c, CA increased to 98.0° for the PDMAEMA grafted sample after the deposition of Ag NPs. For the quaternized membrane, loading of Ag NPs increased the CA from 45.2° to 60.3°.

On the other hand, following the deposition of Ag NPs to the membranes, there WAS a very significant increase in CA, mainly due to increased porosity, in agreement with previous data.

**Figure 6:**
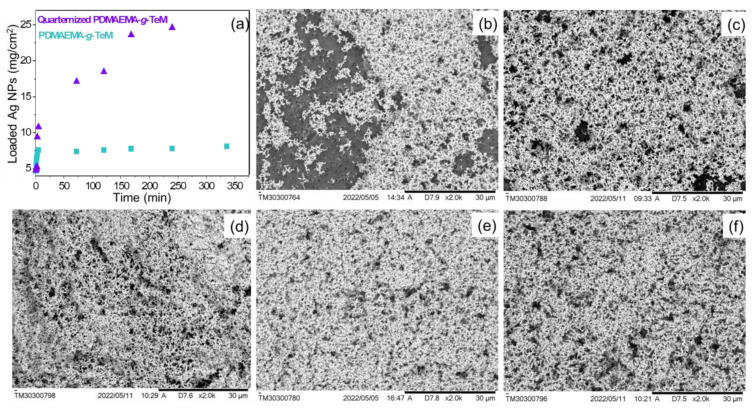
Loading of Ag NPs onto PDMAEMA-*g*-TeM (DG: 22%) and quaternized PDMAEMA-*g*-TeM of the same DG (**a**). SEM images of PDMAEMA-*g*-TeM (DG: 22%) surface after 30 min (**b**), 5 h (**c**), and 24 h of Ag NPs loading (**d**). SEM images of quaternized PDMAEMA-*g*-TeM surface (same DG) after (**e**) 2 h and 24 h of Ag NPs loading (**f**).

**Figure 7:**
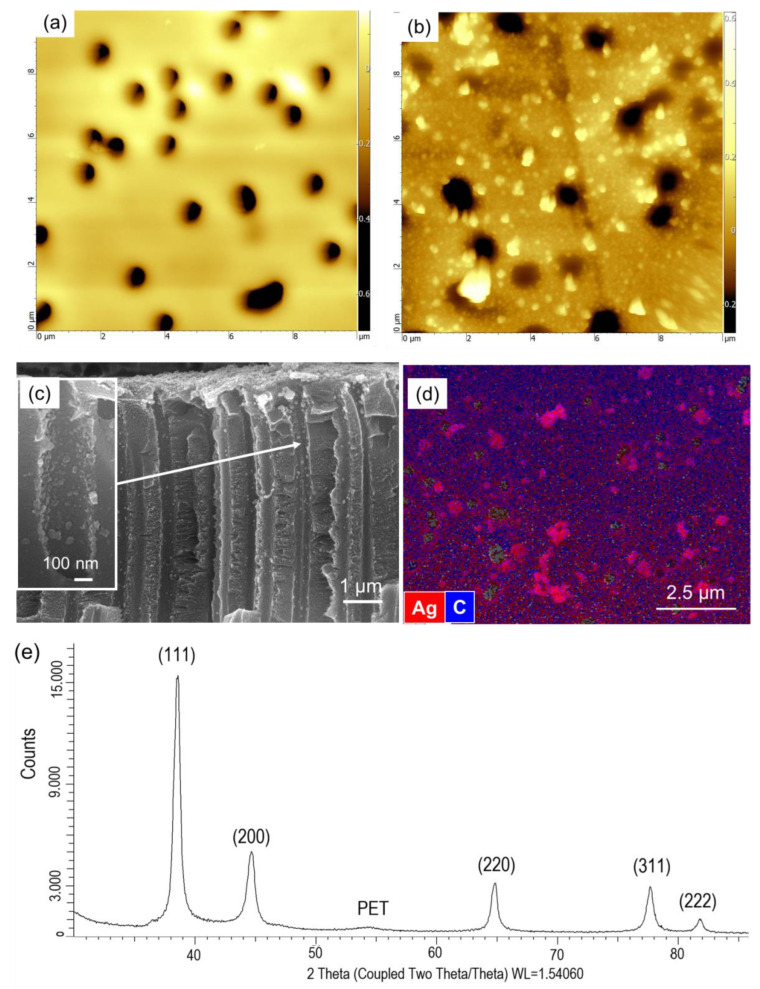
Atomic force microscopy (AFM) image of PDMAEMA-*g*-PET (**a**) and Ag@PDMAEMA-*g*-PET (**b**). The scanning area was 10 × 10 µm^2^. Cross-sectional SEM image (**c**), SEM-EDX surface elemental mapping (**d**) and X-Ray diffraction (XRD) pattern of Ag loaded PDMAEMA-*g*-PET (Ag@PDMAEMA-*g*-PET) (**e**).

The tertiary amine groups of PDMAEMA grafts were then chemically treated with dimethyl sulphate to obtain quaternized PDMAEMA chains having high ion-exchange properties. Quaternization transforms PDMAEMA into a strong polyelectrolyte [80] with promising arsenic removal capacity [53] and antimicrobial properties [81,82] that may be notable for simultaneous disinfection of drinking water. Another obvious advantage of quaternization appeared in Ag NPs loading study. As can be seen from Figure 6a, the amount of Ag NPs loaded onto the quaternized membrane was quite high compared with that obtained for the PDMAEMA-grafted one. In both types of membranes, after a fairly rapid period of rise, equilibrium was observed. The equilibrium was reached after about 5 h of loading in PDMAEMA-grafted membrane. SEM images (Figure 6b–d) also confirm the increased amount of Ag NPs and the achievement of the equilibrium for the PDMAEMA-*g*-TeM sample. For the quaternized sample, the time to reach equilibrium was longer, but the final loading amount was higher. Despite the seemingly pleasant advantages, quaternization caused a fatal problem. The quaternized membranes were quite fragile. Therefore, it was completely abandoned from working with quaternized samples, especially since they pose a serious problem in terms of maintaining their physical integrity in long-term or repeated use, and As(III) removal performances were examined employing only PDMAEMA-grafted and Ag NPs-loaded samples (Ag@PDMAEMA-*g*-PET). Atomic force microscopy (AFM) was also used to show the presence of Ag NPs on PDMAEMA-*g*-PET. Figure 7a depicts the typical surface morphology of PDMAEMA-*g*-PET where only nanochannels are displayed on the sample surface. On the other hand, Ag@DMAEMA-*g*-PET membrane surface exhibited Ag clusters of different sizes approximately in the range of 40–250 nm (Figure 7b). It is worth noting that the membrane surface is completely covered with Ag NPs by increasing the loading time, as can be seen from the SEM images in Figure 5b–f. In addition to covering the entire surface, Ag NPs are also present inside the nanochannels, as can be seen in the cross-sectional SEM image in Figure 6c. Although Ag NPs are stacked at the nanochannel entrances, we predict that the As(III) solution can easily leak through the gaps between the nanoparticles, and thus the solution can reach the PDMAEMA grafted nanochannels. Homogeneous surface coverage of the TeM surface with Ag NPs was also demonstrated by SEM-EDX element mapping as shown in Figure 7d. The X-ray diffractogram (XRD) of the Ag loaded PDMAEMA-*g*-PET in Figure 7e identified Ag phase-specific diffraction peaks at 2θ = 38.43° (111), 44.64° (200), 64.65° (220), 77.60° (311), and 81.82° (222). The defined planes are in accordance with the Crystallography Open Database (COD) file (COD: 01509146) corresponding to cubic structure of Ag (symmetry group Fm-3m(225)). The average size of the loaded silver nanoparticles was calculated as 17.7 ± 3.5 nm using the Scherrer equation. The degree of crystallinity was 78.62%.

### 3.2. Kinetic Study of As(III) Removal

**Figure 8:**
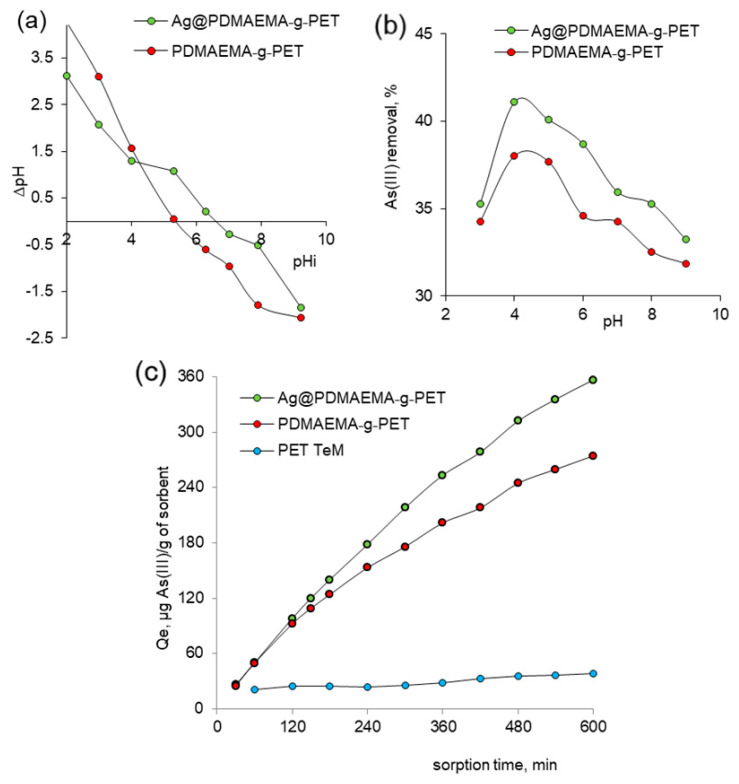
Point zero charge (pH_PZC_) plot (**a**), effect of pH on As(III) removal (contact time: 300 min, As(III) concentration: 50 ppm) (**b**), effect of contact time on As(III adsorption capacity (pH: 4.0, As(III) concentration: 50 ppm) (**c**).

The pH of adsorption medium is one of the most critical factors for evaluating the removal efficiency of the analyte and the suitability of an adsorption system in real applications [83]. The pH of zero point (pH_PZC_) corresponds to the pH at which the positive and negative charges on the surface of an adsorbent are equal [84]. pH_PZC_ was calculated from the plot of the initial and final pH values (Figure 8a) and determined as 5.3 and 6.7 for PDMAEMA-*g*-PET and Ag@PDMAEMA-*g*-PET, respectively. At pH values lower than pHpzc, the composite will be positively charged, while it will have a net negative charge if pH > pH_PZC_. This parameter is important for the heavy metal adsorption process because at values higher than pH_PZC_, the surface of adsorbents will be negatively charged, reducing the electrostatic interactions between As(III) oxyanions and TeM surface [85]. The effect of pH on the adsorption of As(III) was investigated in the pH range of 2.0 to 8.0 (Figure 8b). Between pH 3.0 and 4.0, As(III) removal efficiency increased from 35.3% to 41.1% for Ag@PDMAEMA-*g*-PET. Further increase in pH (5.0–9.0) resulted in a decrease in sorption capacity, and the removal efficiency was only 33.2% at pH 9.0. A similar trend was seen with the use of PDMAEMA-*g*-PET, and thus the optimal pH value of As(III) feed solutions was chosen as 4.0 in all subsequent batch adsorption experiments. In order to elucidate the adsorption kinetics, the kinetic sorption curves in Figure 8c were plotted. It was determined that the adsorption reached equilibrium in approximately 540 min and the As(III) removal efficiency after 10 h was around 85.6% and 56% for Ag@PDMAEMA-*g*-PET and PDMAEMA-*g*-PET, respectively. In order to exclude the effect of the PET template on the sorption capacity of the prepared composites, pristine PET TeM was also examined in As(III) sorption. As can be seen in Figure 8c, the PET template itself had a very low arsenic sorption capacity of 17.5%, clearly demonstrating the effect of the modifications carried out on As(III) removal.

**Figure 9:**
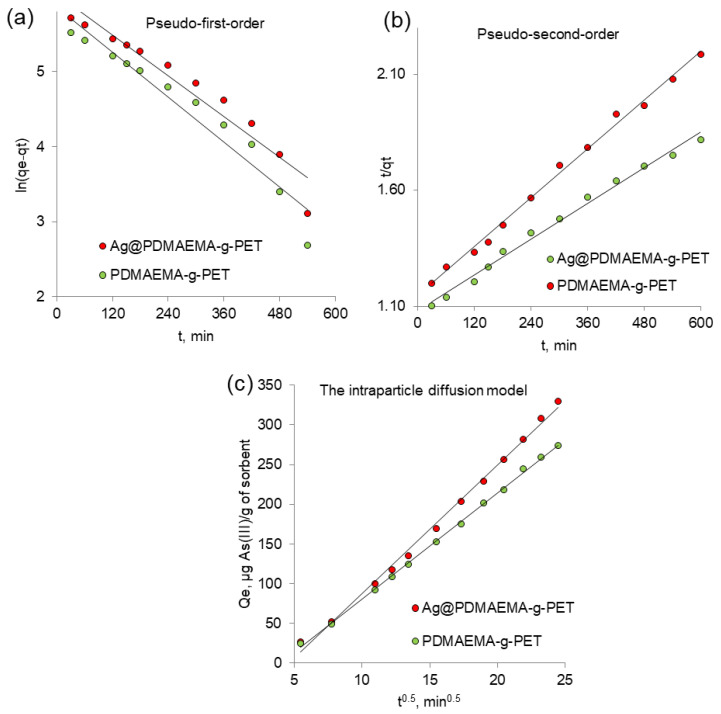
Kinetics of As(III) adsorption according to the studied kinetics models: pseudo-first order (**a**), pseudo-second-order (**b**) and the intraparticle diffusion model (**c**).

The adsorption rate is one of the most valuable criteria that determine the effectiveness of the adsorbent. The possible rate controlling step, as well as the mechanism of adsorption, can be deduced from the study of adsorption kinetics [86]. In this study, linear and nonlinear form of pseudo-first and pseudo-second-order kinetics, as well as intra-particle diffusion model, were applied on adsorption data. Figure 9a–c show the kinetic plots for the models studied. Correlation coefficients (R^2^), linearized equations, and parameters calculated from these models are summarized in Table 1. The data for both samples fit well with the pseudo-second-order model, confirming the literature data on the adsorption mechanism of As(III) by other composite sorbents such as Cu@PET composite TeMs [35], zeolite-reduced graphene oxide composite [87], and copper oxide-incorporated mesoporous alumina [88]. Intraparticle diffusion model was studied to understand whether the transport and diffusion of adsorbate is a rate-determining step. Figure 9c indicated the linear feature of the intraparticle model. If the plot of *t*^0.5^ versus *q_t_* gives a straight line, the adsorption process is solely controlled by intraparticle diffusion. In contrast, if the linear fitting exhibits multilinear curves or does not exactly pass through the origin, two or more steps will influence the adsorption process. Since plots on the Figure 9c do not pass through the origin, intraparticle diffusion is not the only rate-controlling step [89]. The mechanism of sorption by adsorbent involves a more complex mechanism including both surface adsorption as well as intraparticle diffusion [90].

### 3.3. Equilibrium Studies of As(III) Adsorption

**Figure 10:**
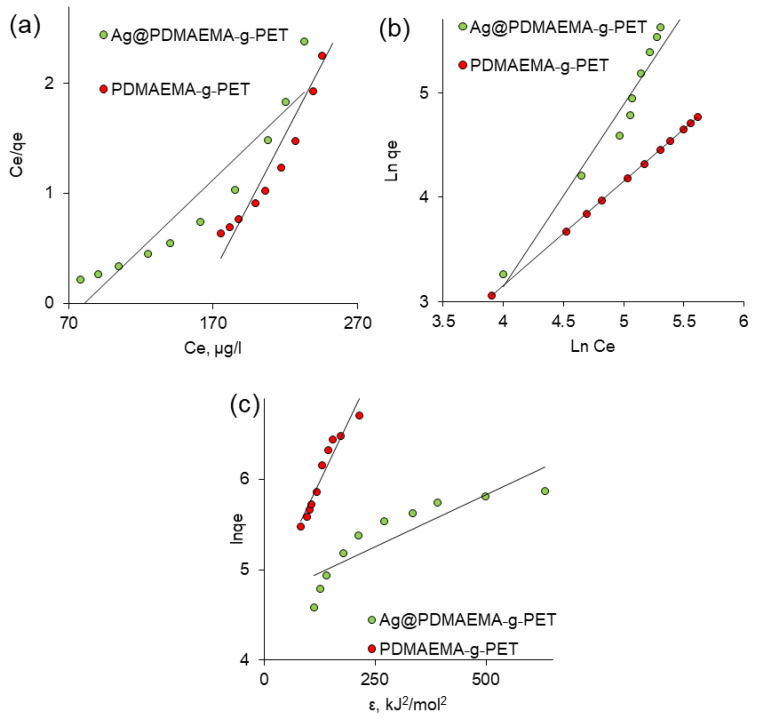
Fitted Langmuir (**a**), Freundlich (**b**), and DR (**c**) adsorption isotherms for the adsorption of As(III).

In order to select the adsorption model that best describes the sorption process, experimental data were fitted with Langmuir, Freundlich and Dubinin–Radushkevich isotherms. The linearized equation forms of all studied isotherm models and the determined isotherm parameters are presented in Table 2. The Langmuir adsorption isotherm describes the surface as homogeneous, assuming no lateral interaction between adjacent adsorbed molecules when a single molecule occupies a single surface site [91]. Langmuir parameters b and $Q_{0}$ were calculated from the intercept and slope, respectively, of the linear plot presented in Figure 10a.

The Freundlich isotherm is one of the most frequently used isotherm models in the description of heterogeneous systems. It assumes neither homogeneous energy sites nor limited adsorption levels. This means that the Freundlich model can describe the experimental data of the adsorption isotherm whether adsorption takes place on homogeneous or heterogeneous sites and is not controlled by monolayer formation. According to this model, the adsorption centers have different energies; therefore, the active sorption sites with maximum energy were filled first followed by the others [92]. Figure 10b shows experimental data adapted to the linear Freundlich equation. The adsorption intensity constant “*n*” is an empirical parameter related to the adsorption strength, which varies depending on the heterogeneity of the adsorbent. In our study, value of n was calculated as 0.64 and 1.0 for Ag@PDMAEMA-g-PET and PDMAEMA-g-PET, respectively. From the parameters in Table 2, it can be said that the Freundlich model best fits the experimental adsorption equilibrium data of As(III) on Ag@PDMAEMA-g-PET and PDMAEMA-g-PET TeMs, indicating that the adsorption process occurs in the form of multilayers with different adsorption energies and non-uniform heat distribution, due to the heterogeneity of the active centers of the adsorbents. The ions first occupied active adsorption sites that present strong interactions, thereby decreasing the interactions [93]. The $k_{F}$ value was higher when As(III) adsorption took place on Ag@PDMAEMA-g-PET, indicating a higher selectivity than DMAEMA-g-PET. The Freundlich’s constant of n = 1.0 calculated for the DMAEMA-g-PET sample indicates that the interactions between adsorbent and As(III) ions were strong, as previously shown, due to the high activity of As(III) and the adsorption capacity of PDMAEMA [53,72]. Increasing the amount of PDMAEMA grafting degree above the optimum value caused closure of nanochannels and deterioration of mechanical properties. Therefore, it should be underlined that loading Ag NPs to the TeMS at the optimum PDMAEMA grafting degree allows for a further increase in the As(III) removal capacity.

The equilibrium experimental data were also tested using the Dubinin–Radushkevich (DR) isotherm model to examine the mechanism of the adsorption process. The DR isotherm model (Figure 10c) is often used to identify physical or chemical adsorption, as the DR constant β is used to determine the sorption energy [94]. The importance of determining the value of $E_{DR}$ stems from the fact that its numerical value can be used to gain insight into the nature of the interactions between As(III) and active centers on the composite surface. When $E_{DR}$ was between 8 and 16 kJ/mol, the process proceeded via ion exchange, and chemisorption was observed at $E_{DR}$ values in the range of 20–40 kJ/mol [95]. The $E_{DR}$ parameter of the DR model was higher for Ag@PDMAEMA-g-PET, indicating that the ion exchange process was higher in TeMs loaded with Ag NPs than in solely PDMAEMA grafted film. The data given in Table 2 show that removal of As(III) by Ag@PDMAEMA-g-PET adsorbent occurs by an ion-exchange mechanism. In case of DMAEMA-g-PET, an adsorption energy of 6.93 kJ/mol was obtained, indicating the presence a process based on physical adsorption. However, a very low R^2^ value (0.79) indicates that the experimental data obtained for Ag@PDMAEMA-g-PET poorly fit the DR adsorption isotherm model and therefore the values obtained may fall outside the confidence interval. A high R^2^ value of the DR isotherm of PDMAEMA-g-PET (0.93) confirms the physical nature of As(III) sorption by grafted TeM.

Many adsorbents have been developed for As(III) removal in the literature. While these materials offer a wide range of results, they have their own advantages and disadvantages. TeMs-based composite membranes have distinct inherent advantages in terms of ease of preparation and application. Table 3 compares the As(III) removal performances of some composite adsorbents available in the literature with the membranes we developed. It should be noted that it is rather difficult to directly compare the present study with previous ones, since the experimental parameters of the sorption such as the amount of sorbent loaded, agitation speed, pH, and sorption temperature are not exactly the same. However, it can easily be said that our results compete closely with existing alternatives and the obtained composite membranes are promising candidates, especially considering the ease of application and high surface area.

## 4. Conclusions

In this work, RAFT-mediated grafting of PDMAEMA from the etched nanochannel walls and surface of the PET TeM was achieved via the UV-activation of benzophenone. Immobilization of PDMAEMA to the entire surface provided a functional absorbent for As(III) ions and stabilized the Ag NPs loaded in the subsequent step. UV-initiated grafting of PDMAEMA was studied at various monomer concentrations, reaction times, and solvents. RAFT-mediated grafting of PDMAEMA from PET-TeMs in water yielded DGs less than 35% for up to 255 min, while a controlled grafting fashion could only be obtained in acetone-water mixture (1:1), where high DG values up to 131% and linearity in DG by reaction time were attained. The grafting of PDMAEMA was followed gravimetrically and PDMAEMA-*g*-PET membranes were characterized by FTIR, XPS, TGA, and SEM. The SEM results revealed that the polymerization took place from the pore walls and the entire surface of PET TeMs, and the optimal grafting degree at which the nanochannels did not close was around 20%. Higher grafting degrees were avoided as the nanopores were closed and the mechanical properties deteriorated, while a further increase in As(III) removal capacity was obtained by Ag NPs loading. Although quaternization of PDMAEMA grafts also increased As(III) removal capacity, it was not performed as the membranes became fragile. SEM-EDX elemental mapping revealed the homogeneity of grafting and Ag NP loading and the presence of Ag NPs inside the nanochannels, while XRD showed a degree of crystallinity of 78.62%. The removal of As(III) by the developed adsorbents was investigated in the pH range of 2.0 to 8.0 and at various sorption times. At the optimal pH value of 4.0, the As(III) removal efficiency after 10 h was 85.6% and 56.0% for Ag@PDMAEMA-g-PET and PDMAEMA-g-PET, respectively, while that of the PET template was very low. Therefore, both PDMAEMA and Ag NPs loading caused a significant increase in As(III) removal. Ag@PDMAEMA-*g*-PET and PDMAEMA-*g*-PET TeMs were found to follow the Freundlich mechanism and a pseudo-second-order kinetic model. Overall, the practicality, finely-tuned functionality, and large surface area of the developed TeMs make them promising sorbent candidates for As(III) removal.

## Figures and Tables

**Table 1:** Parameters calculated from various kinetic models (initial As(III) concentration: 50 ppm, pH: 4.0).

Kinetic Model	Linearized Equation	Model Parameters	Value	
			Ag@DMAE- MA-*g*-PET	DMAEMA-*g*-PET
Pseudo-first-order	$ln\left(q_{e}-q_{t}\right)=ln\;q_{e}-k_{1}t$	$k_{1}$, min^−1^	0.007	0.005
		$q_{e}$, mg/g	762.7	349.6
		R^2^	0.74	0.94
Pseudo-second-order	$\frac{t}{q_{t}}=\frac{1}{k_{2}q_{e}^{2}}+\frac{t}{q_{e}}$	$k_{2}\times 10^{-6}$, g/mg × min	1.50	2.82
		$q_{e,}$, mg/g	769.2	555.6
		R^2^	0.99	0.99
Intra-particle diffusion	$q_{t}=K_{p}t^{0.5}\;$+ *C*	$K_{p}$, mg/(g × h^0.5^)	16.29	13.38
		C, mg/g	75.12	53.3
		R^2^	0.99	0.99

**Table 2:** Parameters calculated from various isotherm models (initial As(III) concentration: 50 ppm, pH: 4.0).

Isotherm Model	Linearized Equation	Model Parameters	Value	
			Ag@ DMAEMA-*g*-PET	DMAEMA -*g*-PET
Langmuir	$\frac{C_{e}}{q_{e}}=\frac{C_{e}}{Q_{0}}+\frac{1}{Q_{0}b}$	$Q_{0}$, µg/g	79.37	39.84
		*b*, L/mg	77.85	10.02
		R^2^	0.89	0.92
Freundlich	$lnq_{e}=lnk_{F}+\frac{1}{n}lnC_{e}$	$k_{F}$, µg/g	18.46	2.84
		n	0.64	1.00
		R^2^	0.99	1.0
Dubinin–Radushkevich	$lnq_{e}=lnQ_{d}-\beta \varepsilon^{2}$	*Q_d_*, µg/g	107.39	107.28
		β, mol^2^/kJ^2^	0.002	0.010
		$E_{DR}$, kJ/mol	14.74	6.93
		R^2^	0.79	0.93

**Table 3:** Comparative adsorption capacity of the prepared TeMs and other composite sorbents towards the adsorption of As(III).

Composite Adsorbent	Sorption Conditions		Qe, mg/g	Ref.
	Initial Concentration of Adsorbate, ppm	Amount of Adsorbent Utilized, mg		
TiO_2_-impregnated chitosan bead	100.0	25.0	2.1	[96]
α-Fe_2_O_3_–polymer monolith	4000.0	500.0	2.7	[97]
Aluminum doped manganese copper ferrite/polymer composite	0.2	50.0	0.053	[98]
Cu/PET TeM	50.0	3.8	0.52	[35]
Cu/Ox_PET TeM		4.0	0.80	
Ag@PDMAEMA-*g*-PET	50.0	9.0	0.357	This study
PDMAEMA-*g*-PET	50.0	6.4	0.274	

## Data Availability

The data presented in this study are available on request from the corresponding author.

## Supplementary Materials

The following supporting information can be downloaded at: https://www.mdpi.com/article/10.3390/polym14194026/s1, Figure S1: UV-Vis spectra of RA1 RAFT agent before and after to exposure to UV-irradiation (a). Pictures taken at the 30th min of UV-initiated, RAFT-mediated grafting of PDMAEMA from PET-TEMs in water (left) and acetone:water (right) (b). Thermal degradation profiles of pristine PET TeM (c) and PDMAEMA grafted PET TeMs with different degrees of grafting of 12% DG (d), 22% DG (e), 29% DG (f), %35 DG(g), and 43% DG (h). Figure S2. Digital camera images of pristine PET TeM (a) and PDMAEMA grafted PET TeMs with degrees of grafting of 12% DG (b), 22% DG(c), 29% DG (d), 35% DG (e), and %43 DG (f). Figure S3. Contact angle (CA) measurements of (a) PDMAEMA grafted TeMs with DG of 22%, its quaternized membrane (b), PDMAEMA grafted TeMs (DG: 22%) with loaded Ag NPs (c), Q-PDMAEMA grafted TeMs (DG: 22%) with loaded Ag NPs (d). Table S1. UV-initiated, RAFT-mediated grafting of DMAEMA from PET-TEMs in water, ethanol, ethanol:water (1:1. *v*/*v*) and acetone:water (1:1. *v*/*v*), [DMAEMA]/[RA1] = 500.

## Abbreviations

**TeM**	**track-etched membrane**
PET	Poly(ethylene terephthalate)
DMAEMA	2-(dimethyamino)ethyl methacrylate
PDMAEMA	poly-2-(dimethyamino)ethyl methacrylate
PDMAEMA-*g*-PET	poly(2-(dimethyamino)ethyl methacrylate) grafted PETtrack-etched membranes
Ag@PDMAEMA-*g*-PET	poly(2-(dimethyamino)ethyl methacrylate) grafted track-etched membranes loaded with silver nanoparticles
RA1	O-ethyl-S-(1-methoxycarbonyl) ethyl dithiocarbonate
TGA	thermogravimetric analysis
XRD	X-ray diffraction
XPS	X-ray photoelectron spectroscopy
SEM	scanning electron microscopy
EDX	energy dispersive X-ray analysis
RDRP	reversible-deactivation radical polymerization
RAFT	reversible addition fragmentation chain transfer polymerization
CTAs	chain transfer agents
BP	benzophenone
pHPZC	pH of zero point
NPs	nanoparticles
Qe	amount of As(III) adsorbed by the unit mass of copper (mg/g)
*C_0_*	feed As(III) concentration (mg/L)
*C* *_e_*	concentration of As(III) in aliquots (mg/L)
DC	degree of crystallinity (%)
L	average crystallite size (nm)
$q_{t}$	adsorption capacity at time t (mg/g)
$k_{1}$	First-order reaction rate constant (min^−1^)
$k_{2}$	pseudo-second-order rate constant of adsorption ($\frac{g}{\mathrm{mg}\times \min}$)
α	initial rate of the adsorption process, mg/g × min
β	desorption constant (g·mmol^−1^)
Ra	roughness of the composite (nm)
b	constant related to the energy of adsorption (i.e., Langmuir constant (L/µg))
*Ce*	equilibrium concentration of adsorbate (mg/L)
$Q_{0}$	maximum monolayer coverage capacity (mg/g)
$k_{F}$	Freundlich isotherm constant related to the adsorption capacity (µg/g)
$Q_{d}$	adsorption capacity of the Dubinin–Radushkevich monolayer (µg/g)
β	constant associated with the free energy of sorption (mol^2^/kJ^2^)
$E_{DR}$	free energy of adsorption (kJ/mol)
