# Supplementary material for: Hybrid PET Track-Etched Membranes Grafted by Well-Defined Poly(2-(dimethylamino)ethyl methacrylate) Brushes and Loaded with Silver Nanoparticles for the Removal of As(III)
Source: Polymers (Basel). 2022 Sep 26;14(19):4026. doi: 10.3390/polym14194026 (PMC9570698; doi:10.3390/polym14194026)
Supplement: Supplementary file 1 [file polymers-14-04026-s001.zip › polymers-1918860-supplementary.pdf]

polymers

MDPI

*Supplementary information*

# Hybrid PET Track-Etched Membranes Grafted by Well-Defined Poly(2-(Dimethylamino)Ethyl Methacrylate) Brushes and Loaded With Silver Nanoparticles for the Removal of As(III)

**Nursanat Parmanbek[1,2], S. Duygu Sütekin[3], Murat Barsbay[3], Anastassiya A. Mashentseva[1,2*], Dmitriy A. Zheltov[1], Nurgulim A. Aimanova[1], Zhanar Ye. Jakupova[2] and Maxim V. Zdorovets[1,4]**

[1] The Institute of Nuclear Physics of the Republic of Kazakhstan, 050032 Almaty, Kazakhstan
[2] Department of Chemistry, L.N. Gumilyov Eurasian National University, 010008 Nur-Sultan, Kazakhstan
[3] Department of Chemistry, Hacettepe University, 06800 Ankara, Turkey
[4] Engineering Profile Laboratory, L.N. Gumilyov Eurasian National University, 010008 Nur-Sultan, Kazakhstan
* Correspondence: a.mashentseva@inp.kz

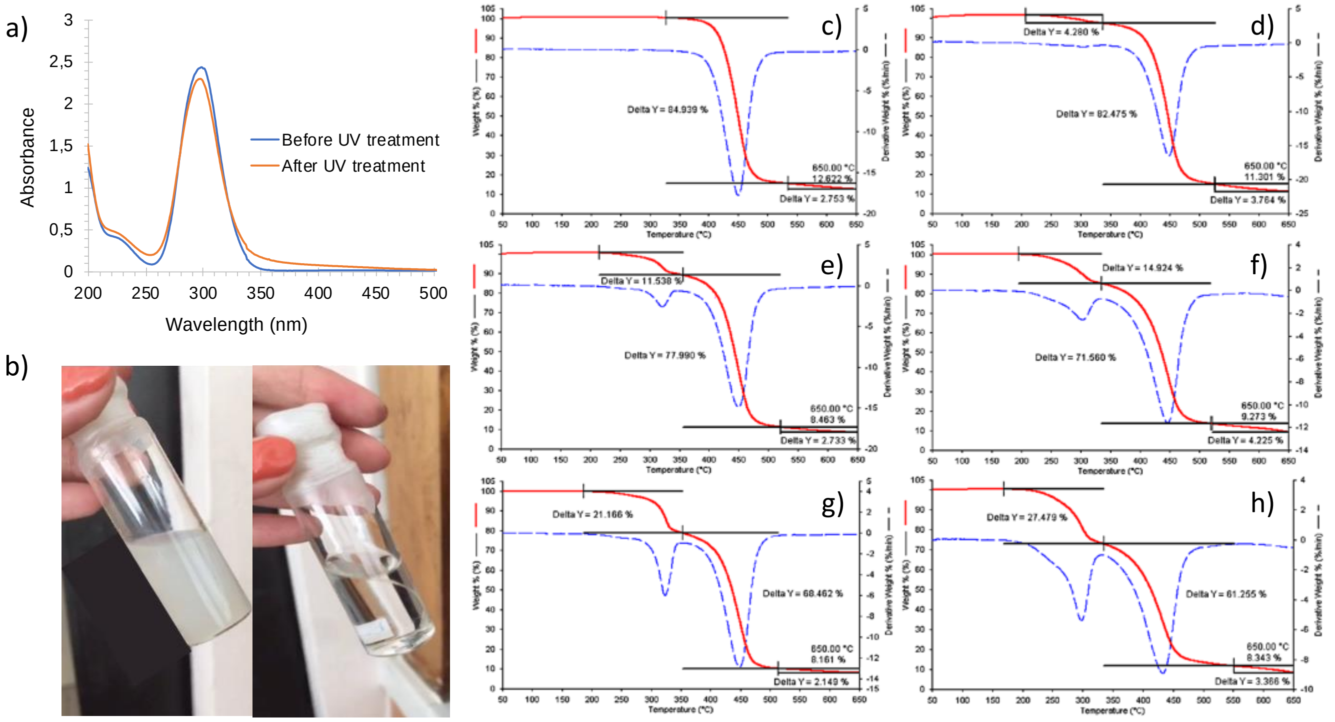


**Figure S1.** UV-Vis spectra of RA1 RAFT agent before and after to exposure to UV-irradiation **(a)**. Pictures taken at the 30th min of UV-initiated, RAFT-mediated grafting of PDMAEMA from PET-TEMs in water (left) and acetone:water (right) **(b)**. Thermal degradation profiles of pristine PET TeM **(c)** and PDMAEMA grafted PET TeMs with different degrees of grafting of 12% DG **(d)**, 22% DG **(e)**, 29% DG **(f)**, %35 DG**(g)**, and 43% DG **(h)**.

**Table S1.** UV-initiated, RAFT-mediated grafting of DMAEMA from PET-TEMs in water, ethanol, ethanol:water (1:1. v/v) and acetone:water (1:1. v/v), [DMAEMA]/[RA1]=500.

| Entry No.. | DMAEMA /RA1 | DMAEMA, v/v % | Solvent | Time (min) | Degree of Grafting (%) |
|---|---|---|---|---|---|
| 1 | 500 | 20 | water | 30 | 5.5 |
| 2 | 500 | 20 | water | 35 | 6.1 |
| 3 | 500 | 20 | water | 40 | 6.8 |
| 4 | 500 | 20 | water | 45 | 7.9 |
| 5 | 500 | 20 | water | 50 | 9.5 |
| 6 | 500 | 20 | water | 55 | 11.8 |
| 7 | 500 | 20 | water | 60 | 14.1 |
| 8 | 500 | 20 | water | 65 | 16.2 |
| 9 | 500 | 20 | water | 135 | 19.7 |
| 10 | 500 | 5 | water | 30 | 2.0 |
| 11 | 500 | 10 | water | 30 | 3.8 |
| 12 | 500 | 30 | water | 30 | 9.3 |
| 13 | 500 | 40 | water | 30 | 12.0 |
| 14 | 500 | 10 | water | 45 | 8.3 |
| 15 | 500 | 10 | water | 60 | 8.1 |
| 16 | 500 | 10 | water | 90 | 7.0 |
| 17 | 500 | 10 | water | 120 | 9.7 |
| 18 | 500 | 10 | water | 180 | 6.1 |
| 19 | 500 | 10 | water | 240 | 5.8 |
| 20 | 500 | 20 | ethanol | 50 | 3.0 |
| 21 | 500 | 20 | ethanol:water | 50 | 3.3 |
| 22 | 500 | 20 | acetone:water | 50 | 12.2 |
| 23 | 1000 | 20 | water | 45 | 15.8 |
| 24 | 1000 | 20 | water | 75 | 17.8 |
| 25 | 1000 | 20 | water | 135 | 29.4 |
| 26 | 1000 | 20 | water | 195 | 31.2 |
| 27 | 1000 | 20 | water | 255 | 35.8 |
| 28 | 1000 | 20 | acetone | 45 | 6.0 |
| 29 | 1000 | 20 | acetone:water | 45 | 11.7 |
| 30 | 1000 | 20 | acetone:water | 75 | 29.3 |
| 31 | 1000 | 20 | acetone:water | 135 | 42.9 |
| 32 | 1000 | 20 | acetone:water | 195 | 66.1 |
| 33 | 1000 | 20 | acetone:water | 255 | 81.7 |
| 34 | 1000 | 20 | acetone:water | 375 | 130.8 |
| 35 | - | 10 | acetone:water | 30 | 22.0 |
| 36 | - | 20 | acetone:water | 30 | 34.9 |
| 37 | - | 20 | acetone:water | 60 | 61.9 |
| 38 | - | 20 | acetone:water | 120 | 98.7 |

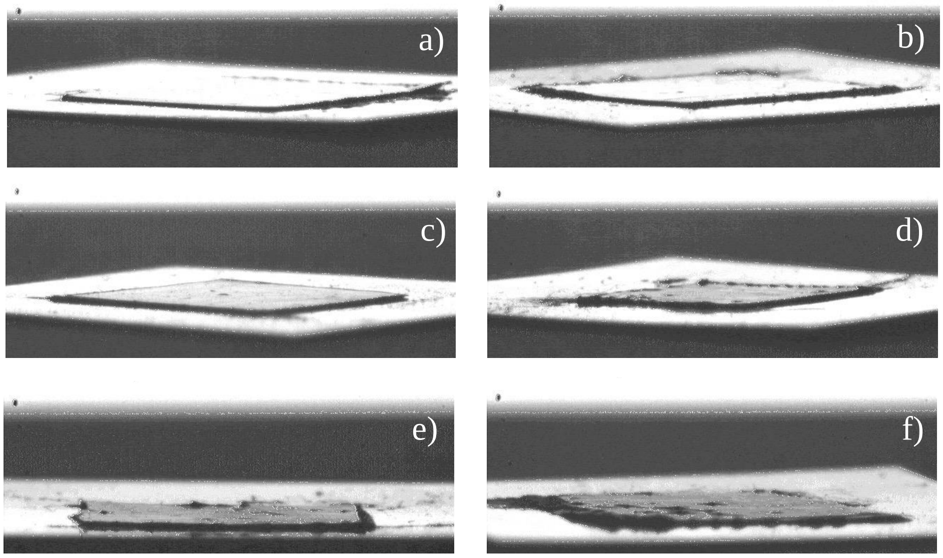


**Figure S2.** Digital camera images of pristine PET TeM **(a)** and PDMAEMA grafted PET TeMs with degrees of grafting of 12% DG **(b)**, 22% DG**(c)**, 29% DG **(d)**, 35% DG **(e)**, and %43 DG **(f)**.

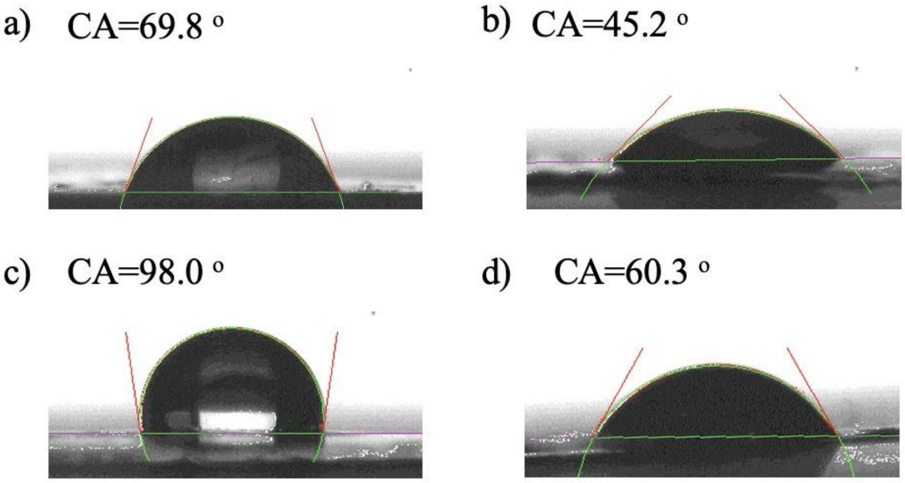


**Figure S3.** Contact angle (CA) measurements of (a) PDMAEMA grafted TeMs with DG of 22%**,** its quaternized membrane (b), PDMAEMA grafted TeMs (DG: 22%) with loaded Ag NPs (c), Q-PDMAEMA grafted TeMs (DG: 22%) with loaded Ag NPs (d).
